# Modified stochastic medium prediction model for the deformation response of concealed underground stations under existing pipelines

**DOI:** 10.1038/s41598-023-37067-3

**Published:** 2023-06-17

**Authors:** Junru Zhang, Tong Pan, Kaimeng Ma, Qiang Xu, Chao Kong

**Affiliations:** 1 School of Civil Engineering, Southwest Jiaotong University, Chengdu, 610031 China; 2 School of Civil Engineering, Shijiazhuang Tiedao University, Shijiazhuang, 050043 China; 3 School of Civil Engineering and Architecture, Southwest University of Science and Technology, Mianyang, 621000 China

**Keywords:** Civil engineering, Applied mathematics

## Abstract

The underground pipeline network in the city is so intertwined that the concealed excavation of a metro station inevitably leads to a series of underground pipelines, causing settlement deformation and further risk of leakage. The existing theoretical methods for analysing settlement deformation are mostly for circular chambers, whereas metro stations have a nearly square cross-sectional form and different construction methods are very different, which have a greater impact on the deformation of the overlying pipelines. In this paper, based on the random medium theory and Peck's formula, the improved random medium model for predicting ground deformation is modified, the correction coefficients *λ* and *η* for the influence of different construction methods are proposed, the prediction model of underground pipeline deformation under different construction methods is obtained, and the numerical models of four work methods commonly used in urban tunnel construction: pillar hole method, side hole method, middle hole method and Pile-Beam-Arch (PBA) method are constructed through simulation, and the mathematical analysis software was used to fit the results to the model and obtain the range of correction coefficients *λ* and* η* for each of the four methods, and the accuracy and applicability of the theoretical model was verified by combining with actual engineering cases. The influence on the overlying pipes is in descending order: side hole method, pillar hole method, middle hole method and PBA method. The theoretical model provided in this paper for predicting the deformation of pipes in any overlying strata of the tunnel is well suited to the actual project and has a high degree of correlation with the measured results.

## Introduction

**Figure 1:**
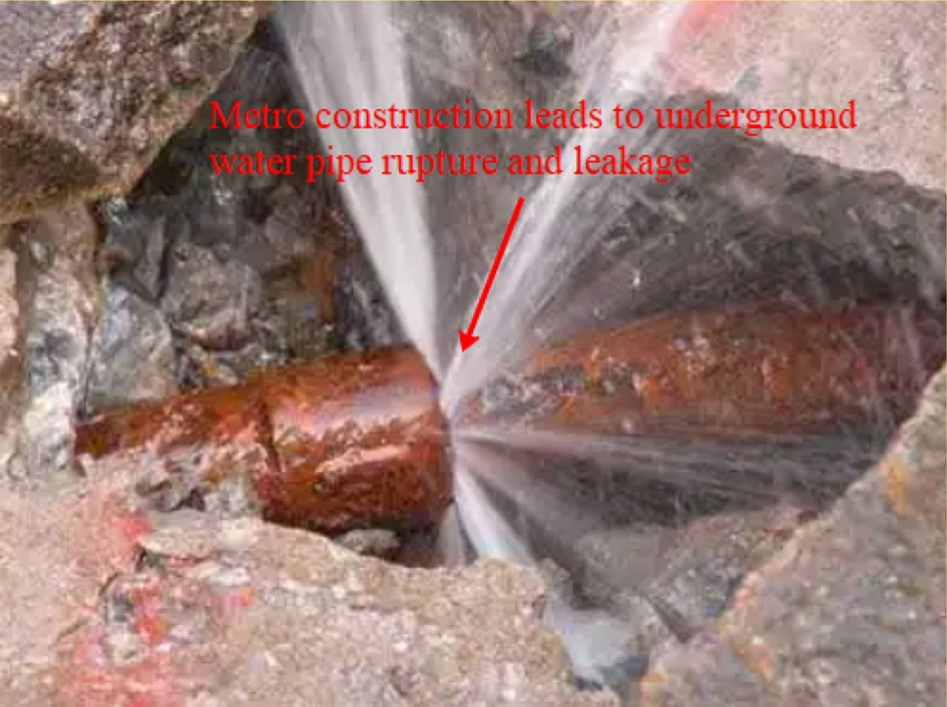
Rupture of water supply pipe due to metro construction.

With the rapid development of metro construction in recent years, various concealed metro station construction methods have been optimised and perfected, resulting in a series of multi-span structure construction methods such as the pillar hole method, the middle hole method, the PBA method and the side hole method, which are suitable for large cross-sections and poor ground conditions^1^. However, most of these methods are complex and disturb the ground, in the context of today's intertwined urban underground pipelines^2^, these features will undoubtedly have a greater impact on the adjacent pipelines, leading to pipeline deformation, cracking and even damage fracture, as shown in Fig. 1

**Figure 2:**
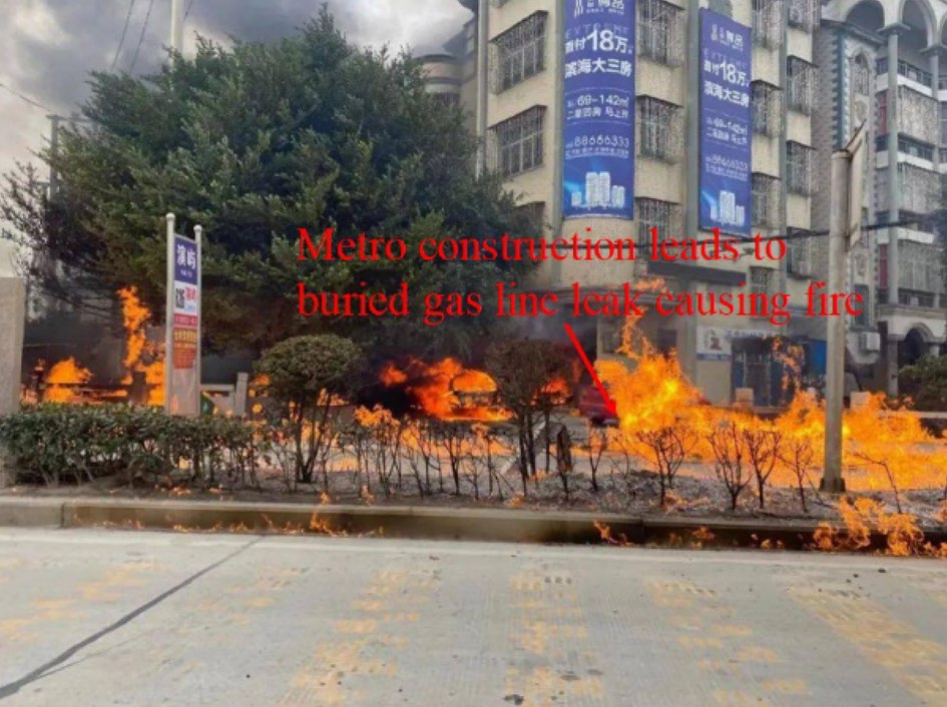
Gas pipe leak causes fire.

Underground pipelines are the lifeline of a city. The basic supply of water, electricity and gas depends on underground pipelines, which can affect the normal life of tens of thousands or even millions of people in case of failure^3–5^. In severe cases, buried gas pipes, once broken, are highly susceptible to leakage, which may lead to serious explosions and pose a great safety threat to surrounding buildings and people^6^.As shown in Fig. 2, the fire was caused by a leak in the pipeline.In addition, damage to underground pipelines and leakage of their contents can cause changes in the surrounding geological conditions, such as local saturation caused by pipeline leakage in areas with wet loess soils, resulting in uneven settlement of foundations and causing safety hazards in the surrounding buildings^7^; At the same time, due to the invisibility of the underground pipelines, their maintenance costs are also high after breakage^8^. Therefore, a good prediction of the deformation of the adjacent pipelines and timely construction response is the key to ensure the successful completion of the project, which has important theoretical and engineering significance for the design and construction of underpass-type tunnels and ensuring the safety of existing underground structures^9–11^.

Currently, investigations into the deformation of adjacent pipelines resulting from metro tunnel construction have employed various methods, such as theoretical analysis, numerical analysis, model tests, analytical methods, and semi-analytical semi-numerical methods to study the deformation of adjacent pipelines under different influencing factors^12^. Among these, theoretical analysis is widely used due to its convenience and high degree of modelling. In this regard, stochastic medium theory and Peck's formula remain the most commonly used methods of analysis and prediction^13^.

The stochastic medium theory was initially proposed by Polish scholar J. Litwinszyn and was later developed by Chinese scholars Liu Baochen and Yang Junsheng^14,15^ for predicting settlement caused by underground tunnel construction. Loganathan, Han Xuan et al.^16–18^ verified the convergence pattern and obtained a non-uniform convergence pattern that was more consistent with actual situations, enhancing the prediction accuracy of the stochastic medium model. In comparative studies, Meng Dan and Li Ning et al.^19,20^ concluded that the Peck formula can be approximately equivalent to the stochastic medium theory approach for small cross-section tunnels with large burial depths. Yang et al.^21^ used inverse analysis to invert 18 ground settlement profiles collected from the literature and pointed out that determining the convergence and the value of the influence angle is crucial to simulating settlement profiles. Xu et al.^22^ simplified and improved the stochastic medium theory based on the relationship between the key parameters of the stochastic medium theory and Peck's formula, and obtained a calculation formula for determining the settlement of overlying arbitrarily buried strata caused by tunnel excavation.

From the extant research, it is evident that numerous experts and scholars have significantly enhanced the random medium theory in predicting the deformation of the ground surface or layer. The deformation mode, essential parameters and factors affecting the theoretical model have been studied and analyzed to varying degrees. However, the research mainly focuses on surface prediction, and while predicting strata deformation, the effects of different construction methods are generally ignored, and the deformation of pre-existing pipelines inside the strata cannot be predicted scientifically. To fill this gap, this paper employs the random medium theory as the foundation, based on a comprehensive review of previous studies, modifies the improved random medium model by means of numerical simulations of commonly used metro station construction methods, including the column hole method, middle hole method, side hole method, and PBA method. This approach enables the development of a pipeline deformation prediction model for neighboring structures under diverse construction methods, and the accuracy and applicability of the model are validated through the analysis of engineering examples.

## Theoretical model

### Stochastic medium theory

The stochastic medium theory originated from the field of mining engineering, where it was initially employed to forecast the displacement of rocks and the ground surface due to mining activities. As time progressed, the theory was gradually introduced to China, and underwent optimization and refinement by a significant number of experts and scholars led by Liu Baochen, resulting in the establishment of a more comprehensive conceptual model.

In the calculations of this theory, the geotechnical body within a certain range of the excavated part is considered as a discontinuous medium. During the excavation process, the medium units are separated from each other, leading to relative motion, which disrupts the original relationship between the units. Based on the probability integration method, the ground movement caused by the excavation process is considered a stochastic process. The excavation of the entire underground structure is then decomposed into infinitely many infinitesimal units. Finally, the impact of the overall excavation on the upper strata is obtained by integrating these infinitely decomposed foundation units, and the profile equation of surface settlement is established through iterative optimization^23^.

From the solution process, it is evident that the stochastic medium theory can capture the impact of different excavation sections on the ground by controlling the integration limits, making it suitable for predicting surface displacement during the construction of various underground structures. In urban underground construction, since most underground tunnels are buried at shallow depths and mostly in the topsoil stratum, the properties of these media are more similar to those of discontinuous media, which can be better represented as random media to enhance the accuracy of model prediction. Hence, random media theory finds extensive use in predicting surface settlement during urban underground construction^24^.

**Figure 3:**
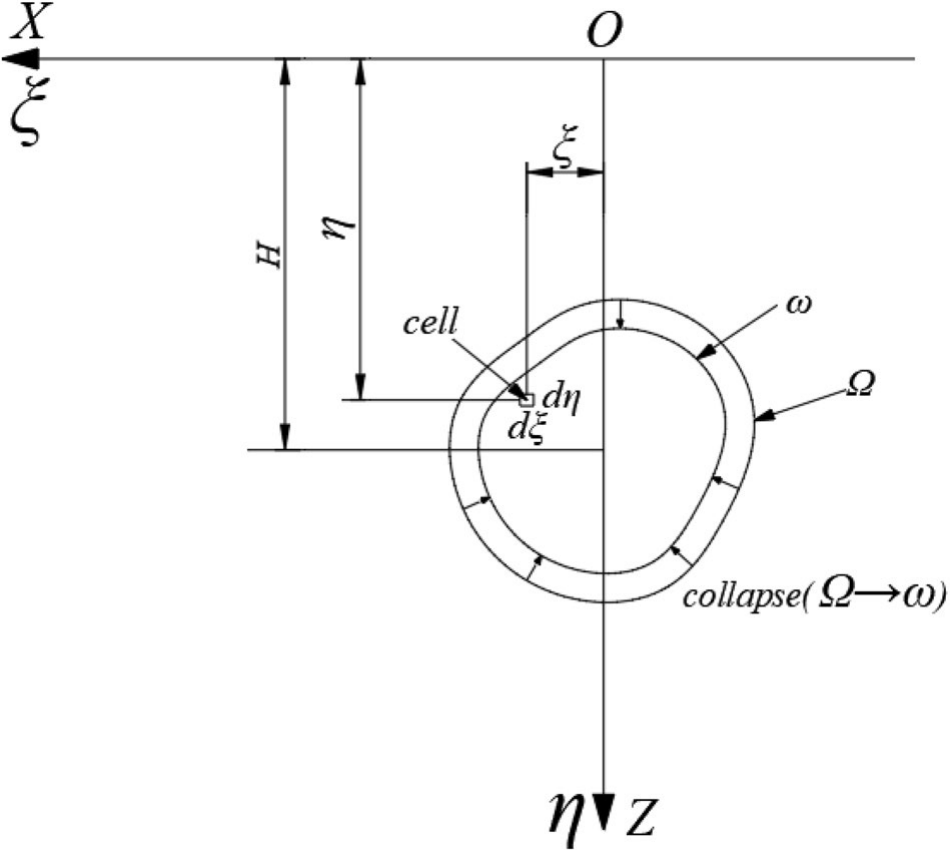
Theoretical model of a random medium.

In general, the longitudinal length of a tunnel is much greater than its cross-sectional dimensions. According to the theory of elasticity, the problem of settlement changes caused by tunnel construction can be simplified into a plane strain problem for studying its laws. While large shallow buried tunnels in soft soil may produce large plastic deformation, which is not applicable to elastic theory. However, when the deformation is small, this difference is acceptable. Therefore, it is scientific and reasonable to simplify the problem of stratum settlement change caused by tunnel construction into plane strain problem^25^ As shown in Fig. 3, an underground tunnel with an arbitrarily shaped section is excavated at a certain depth from the ground, where the center of the tunnel is at a distance of *H* from the surface. The initial section of the excavation is *Ω*, and the section shrinks to *ω* after the tunnel is completed. The figure uses the coordinate system *ξOη* for the geotechnical body of the excavation unit, and the *XOZ* coordinate system for the ground surface.

The final settlement value *We(x)* of the ground surface at a distance* x* from the center of the unit, under the condition of no drainage, no consolidation, and with constant ground density, when the excavated unit has completely collapsed can be expressed as:

1 $$W_{e}^{{}} (x) = \frac{1}{r(z)}\exp \left[ { - \frac{\pi }{{r_{{}}^{2} (z)}}x_{{}}^{2} } \right]d\xi d\eta$$

where *r*(*z*) is the main influence range on the horizontal surface of the unit at a depth of *z*. Its value depends on the stratigraphic conditions in which the tunnel is located, and its relationship with *z* is variable (can be linear or non-linear). Here the angle of main influence of the stratigraphy *β* is introduced, and it is assumed that *r*(*z*) is linear with *z*, i.e. *r*(z) = z/tan*β*.

Applying the principle of superposition, the total surface subsidence W(x) can be calculated as the difference between the subsidence caused by the initial excavation area Ω and the subsidence caused by the final excavation area ω, that is:

2 $$\begin{gathered} W(x) = W_{\Omega }^{{}} (x) - W_{\omega }^{{}} (x) \\ = \iint\limits_{\Omega - \omega } {\frac{\tan \beta }{\eta }}\exp \left[ { - \frac{{\pi \tan_{{}}^{2} \beta }}{{\eta_{{}}^{2} }}(x - \xi )_{{}}^{2} } \right]d\xi d\eta \\ \end{gathered}$$

**Figure 4:**
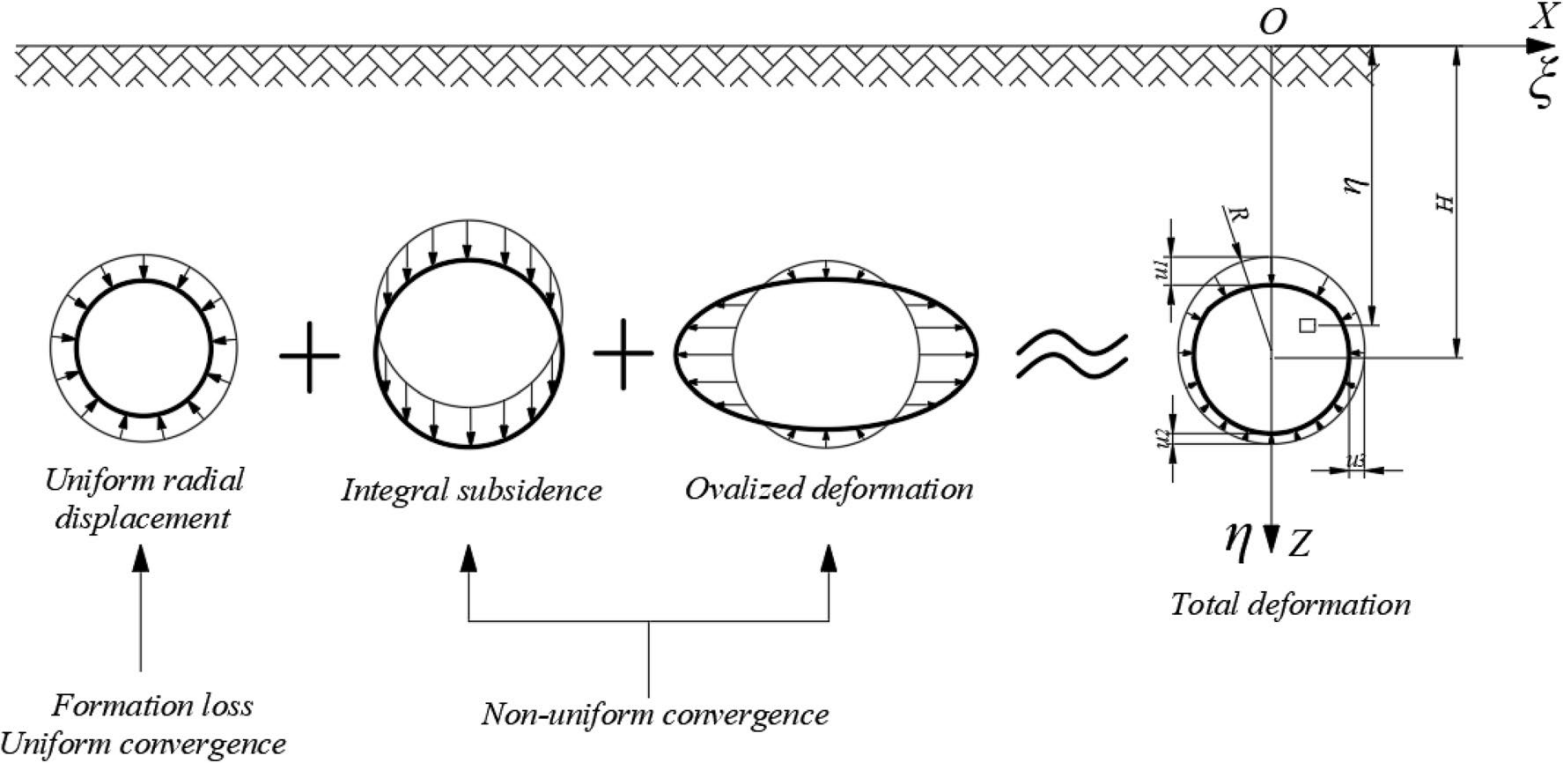
Schematic diagram of the non-uniform convergence calculation model.

Taking into account the non-uniformity of the tunnel convergence pattern, the Eq. (2) is modified by drawing on prior research outcomes^22,26,27^, and is illustrated through a circular tunnel as an instance. An illustration of the computational model for the non-uniform convergence of the tunnel is shown in Fig. 4:

3 $$W(x) = \frac{{\pi R[(u_{1}^{{}} + u_{2}^{{}} )/2 + u_{3}^{{}} ]tan\beta }}{H}\exp \left( { - \frac{{\pi \tan_{{}}^{2} \beta }}{{H_{{}}^{2} }}x_{{}}^{2} } \right)$$

where: *R* is the tunnel excavation radius, *u*_*i*_(i = 1,2,3) is the section convergence value.

### An improved stochastic medium model for calculating ground settlement

**Figure 5:**
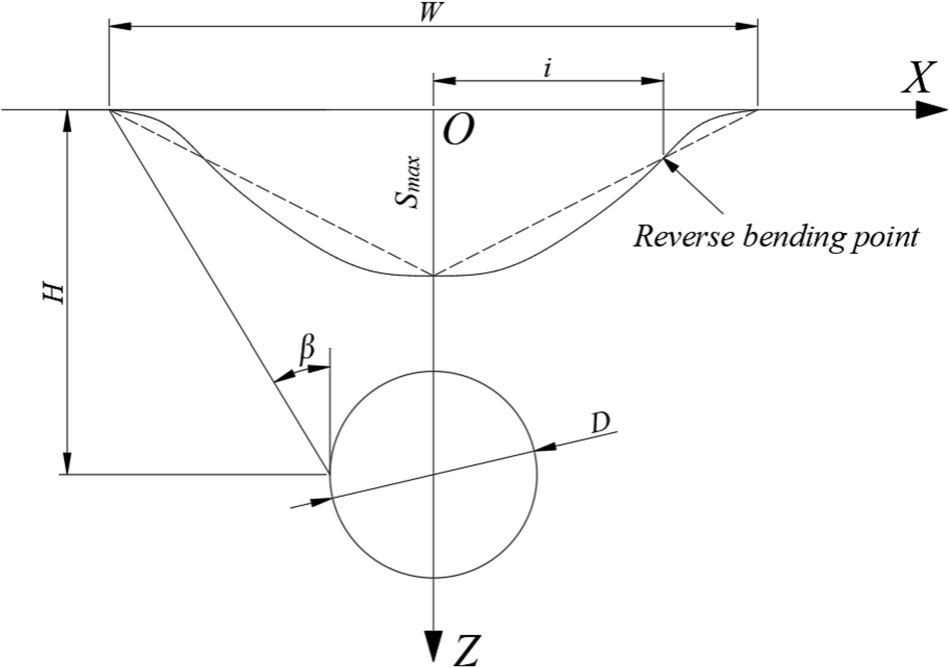
Lateral distribution of settling troughs.

The traditional random medium theory is primarily utilized for predicting and analyzing ground surface settlement. Xuan Han^28^ established a correlation between the random medium model and the depth of soil cover by studying the relationship between the random medium theory and Peck's formula. Furthermore, the variation of displacement at different soil depths under different conditions of the settlement trough width *i* was compared and analyzed. The lateral distribution of the settling troughs obtained based on the Peck formula is shown in Fig. 5.

Observe that the Peck formula:

4 $$S_{\max }^{{}} = \frac{V}{{\sqrt {2\pi } i}}$$

5 $$V = \frac{{V_{l}^{{}} \pi D_{{}}^{2} }}{4}$$

where: *S*_*max*_ is the ground settlement maximum, *V* is the stratigraphic loss per unit length of the tunnel, *V*_*l*_ is the stratigraphic volume loss rate^29^, *D* is the diameter of the tunnel excavation and *i* is the width of the settlement trench.

The Peck formula expresses the strata loss rate *V*_*l*_ as the percentage of the total volume of the excavated part that is the unit length of strata loss. In the random medium theory, the loss of strata corresponds to the section shrinkage of the excavated unit integration, and the two have the same longitudinal length. Therefore, the strata loss rate *V*_*l*_ can be expressed through the change in section shrinkage. The relationship between the two is shown in Eqs. (6) and (7):

6 $$V_{l}^{{}} = \frac{{\pi [R_{{}}^{2} - (R - \Delta R)_{{}}^{2} ]}}{{\pi R_{{}}^{2} }} = \frac{\Delta R(2R - \Delta R)}{{R_{{}}^{2} }}$$

7 $$\Delta R = R(1 - \sqrt {1 - V_{l}^{{}} } )$$

where: *∆R* is the equivalent uniform shrinkage value of the section radius.

The equivalent uniform shrinkage value of the section radius *∆R* can be expressed as a function of the section convergence value *u*_*i*_(i = 1, 2, 3) as shown in Eq. (3). The relationship between the two can be assumed and expressed as shown in Eq. (8):

8 $$\Delta R = f(u_{i}^{{}} )(i = 1,2,3)$$

According to Tong Lei et al^30^, the scaling relationship between the section convergence values shown in Eq. (9) can be obtained using the optimal solution, as follows:

9 $$\begin{gathered} u_{2}^{{}} = \frac{1}{2}u_{1}^{{}} \hfill \\ u_{3}^{{}} = \frac{1}{4}u_{1}^{{}} \hfill \\ \end{gathered}$$

Loganathan and Poulos^16^ investigated the non-uniform convergence of the tunnel section vault convergence value of *2∆R* and proposed a modified formula for the vertical displacement of the strata that fits better with the measured data. The modified formula is given as:

10 $$u_{1}^{{}} = 2\Delta R$$

Substituting Eqs. (4), (5) and (7) into Eq. (10), the collation gives:

11 $$u_{1}^{{}} = 2R\left[ {1 - \sqrt {1 - \frac{{iS_{\max }^{{}} }}{{0.313D_{{}}^{2} }}} } \right]$$

In the random medium theory, the primary influencing angle β of the stratum is correlated with the settlement trough width *i* in Peck's formula, as presented in Eq. (12). Furthermore, to calculate the settlement trough width *i*, Xuan Han^27^ improved the existing formula based on the measured data, as follows:

12 $$tan\beta = \frac{H}{{\sqrt {2\pi } i}}$$

13 $$i = K(H - bz)$$

14 $$K = 1 - 0.02\phi$$

where: *H* is the burial depth of the centre of the tunnel; *K* is the parameter of the width of the sink; *b* is the parameter considering the nature of the stratum, taking values from 0 to 1; *z* is the burial depth of a stratum overlying the tunnel; *ϕ* is the weighted average of the internal friction angle of each stratum above the tunnel vault according to its thickness.

Referring to the literature^22^, the relationship between the random medium theory and the buried position of the strata can be established by substituting Eqs. (13) and (14) into Eqs. (3), (11), and (12). This leads to obtaining a prediction equation for calculating the change in settlement of any overlying strata due to tunnel excavation, as follows:

15 $$W(x) = \frac{{\sqrt {2\pi } R_{{}}^{2} }}{i}\left( {1 - \sqrt {1 - \frac{{iS_{\max }^{{}} }}{{1.252R_{{}}^{2} }}} } \right)\exp \left( { - \frac{{x_{{}}^{2} }}{{2i_{{}}^{2} }}} \right)$$

16 $$i = \left( {1 - 0.02\phi } \right)\left( {H - bz} \right)$$

### Improved stochastic medium model for predicting settlement of adjacent pipelines

Based on previous research, the derivation above was established for a circular tunnel section. However, in urban metro construction, most metro stations have non-circular sections. To enhance the accuracy of the prediction model, Eq. (15) must be modified, where *R* is replaced by the equivalent radius of the tunnel section. Thus, non-circular tunnels can use the value of *R* determined by Eq. (17) to improve accuracy^31,32^:

17 $$R = \sqrt {\frac{A}{\pi }}$$

where: A is the area of the tunnel excavation.

Bringing Eq. (17) into Eq. (15), we have:

18 $$W(x) = \frac{0.798A}{i}\left( {1 - \sqrt {1 - \frac{{2.509iS_{\max }^{{}} }}{A}} } \right)\exp \left( { - \frac{{x_{{}}^{2} }}{{2i_{{}}^{2} }}} \right)$$

Bring Eq. (17) into Eqs. (5) and (6):

19 $$\begin{gathered} V = V_{l}^{{}} .A \hfill \\ S_{{{\text{max}}}}^{{}} = \frac{{V_{l}^{{}} .A}}{{\sqrt {2\pi } i}} \hfill \\ \end{gathered}$$

Taking Eq. (19) into Eq. (18) and simplifying it gives:

20 $$W(x) = \frac{0.798A}{i}\left( {1 - \sqrt {1 - 0.565V_{l}^{{}} } } \right)\exp \left( { - \frac{{x_{{}}^{2} }}{{2i_{{}}^{2} }}} \right)$$

As the pipeline is buried in the strata, and the size of the pipeline is far less than the size of the tunnel, so the model for predicting changes in ground settlement can be used to roughly predict the settlement of the pipeline. But after all, the two are not equivalent, and different construction methods may lead to settlement fluctuations in the pipeline is also larger.To improve the accuracy of pipeline settlement prediction, pending coefficients *λ* and *η* are introduced, and the revised equation is expressed as follows:

21 $$W(x) = \frac{0.798A}{i}\left( {1 - \sqrt {1 - 0.565\lambda V_{l}^{{}} } } \right)\exp \left( { - \frac{{x_{{}}^{2} }}{{2(\eta i)_{{}}^{2} }}} \right)$$

where: *λ* is the *S*_*max*_ correction factor and *η* is the correction factor for *i*.

To determine the correction coefficients for the prediction equations of pipeline settlement under different construction methods, numerical simulation is utilized to simulate the various construction methods. The simulation data is then extracted and used to fit the prediction equations through regression analysis.

## Numerical simulation

### Parameter selection and model building

**Figure 6:**
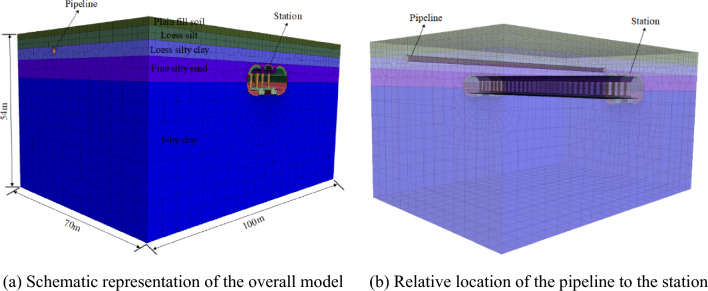
Schematic diagram of the numerical model.

The analysis of surface subsidence caused by some tunnel excavations can be analyzed using numerical methods^33^. Based on an underground pipeline project beneath a metro station, four construction simulation models were established using finite difference software FLAC^3D^. The dimensions of all four models are 100 m × 70 m × 54 m (length × width × height), with the pipeline center located 6.4 m below the surface and the metro station center located approximately 13.9 m below the surface. The pipeline is oriented perpendicular to the main projection of the station, as illustrated in Fig. 6:

The upper surface of the model is free, while the remaining surfaces are constrained to normal displacement. The stratum, primary support, and second lining are all simulated using solid units. The support structure unit adopts the elastic model, while the rest of the stratum materials are simulated using the Mohr–Coulomb model. The parameters for the stratum and structural units are shown in Tables 1 and 2.

**Table 1:** Stratigraphic parameters.

Stratigraphic name	Unit weight (kN/m^3^)	Modulus of elasticity (MPa)	Poisson’s ratio	Cohesion (kPa)	Internal friction angle (°)
Vegetal fill	17.0	10	0.35	15	20
Loess-like chalk	18.7	60	0.32	30	15
Loess-like powdery clay	19.5	72	0.30	38	25
Powdered fine sand	22.0	45	0.30	0	45
Powdery clay	19.7	100	0.28	33	18

**Table 2:** Calculation parameters for structural units.

Name of structure	Unit weight (kN/m^3^)	Modulus of elasticity (MPa)	Poisson's ratio	Cohesion (kPa)	Internal friction angle (°)
Initial support	22	23,240	0.25	–	–
Secondary lining	25	33,500	0.25	–	–

### Construction method introduction and construction sequence

**Figure 7:**
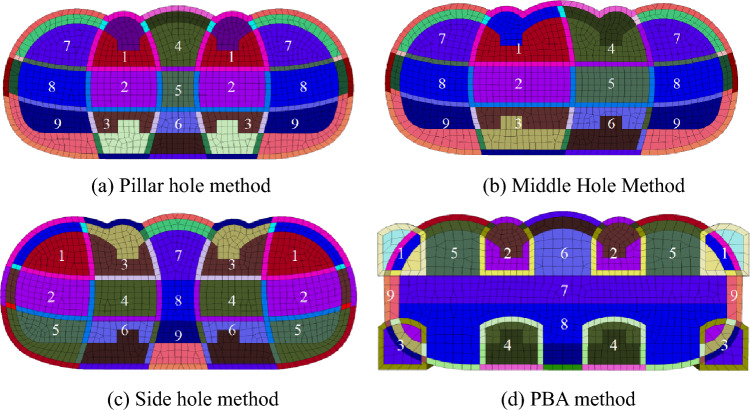
Step-by-step diagram of the four construction methods.

The introduction of the above four construction methods and the construction procedures in numerical simulation are described as follows:

The pillar hole method refers to the construction of a small guide hole at the position of the column, when the small guide hole is completed, the bottom beam is built in the hole to form a thin and high longitudinal structure, and then the soil on both sides of the column is excavated successively to complete the overall construction of the structure, as shown in Fig. 7a. The excavation sequence is: first, excavate the central guide hole (1, 2, 3 in Fig. 7a) and apply the initial and temporary support, then install the beam and column support structure in the central guide hole. Next, excavate the part between the two central guide holes (4, 5, 6 in Fig. 7a) from top to bottom, apply the initial support, remove part of the temporary support, and apply part of the second lining and bottom slab. Then, excavate the guide holes on both sides (7, 8, 9 in Fig. 7a) and apply the initial and temporary support. Finally, remove the temporary support and apply the bottom slab, side walls, and second lining from bottom to top to complete the construction. The closure of the ring is achieved by removing the temporary support and applying the initial support and temporary support, followed by the installation of the bottom slab, side walls, and second lining in order.

Middle hole method refers to a construction method in which the middle part (middle cave) is excavated first, a beam and column structure is constructed in the middle cave, and then both sides (side cave) are excavated, and the load at the top of the side cave is gradually transferred to the beam and column structure through the initial support of the middle cave, as shown in Fig. 7b. The excavation sequence is: first, excavate the central guide hole (1, 2, 3, 4, 5, 6 in Fig. 7b) and apply initial support and temporary support. After stabilization, apply the beam and column support system in the middle hole. Then, excavate the side holes (7, 8, 9 in Fig. 7b) and apply initial support from top to bottom. Finally, remove the temporary support and apply the base slab, side walls, and the remaining second lining from bottom to top to complete the overall construction.

Side hole method refers to a construction method that excavates both sides (side tunnel) first, builds beam and column structure in the side tunnel, and then excavates the middle part (middle tunnel), and gradually transfers the load from the top of the middle tunnel to the beam and column through the initial support, as shown in Fig. 7c. The excavation sequence is: first, excavate the upper and middle parts of the guide cavern on both sides (Fig. 7c 1, 2) and apply initial support and temporary support. Then, excavate the central guide cavern (Fig. 7c 3, 4) and apply initial support and temporary support. Next, excavate the bottom guide cavern in steps (Fig. 7c 5, 6) and apply temporary support and initial support. After excavation, apply the beam and column bearing structure in the central guide cavern. From the bottom to the top in turn, remove the temporary support and apply the base slab, side walls, and upper second lining. Finally, excavate the remaining soil in the centre (7, 8, and 9 in Fig. 7c) and apply the remaining second lining and base slab to close the ring and complete the construction.

PBA method is a kind of underground excavation method, in which a small guide hole is first built, a digging pile is made in the hole, and after the beams-column structure is completed, the top structure is built, and then the subsequent excavation is carried out under the protection of it, as shown in Fig. 7d. The excavation sequence is: first, excavate the small guide hole (1, 2, 3, 4 in Fig. 7d) and apply initial support; set up small guide holes between the upper and lower guide holes, penetrate them and apply beam and column structures; excavate the soil between the guide holes (5, 6 in Fig. 7d), apply initial support, and install the top arch of the second lining; excavate the remaining soil in the middle (7, 8 in Fig. 7d) from top to bottom and apply initial support, side walls, and bottom slab; finally, remove the remaining soil in the center (7, 8 in Fig. 7d) and excess initial support to complete the construction.

### Linear fit

**Figure 8:**
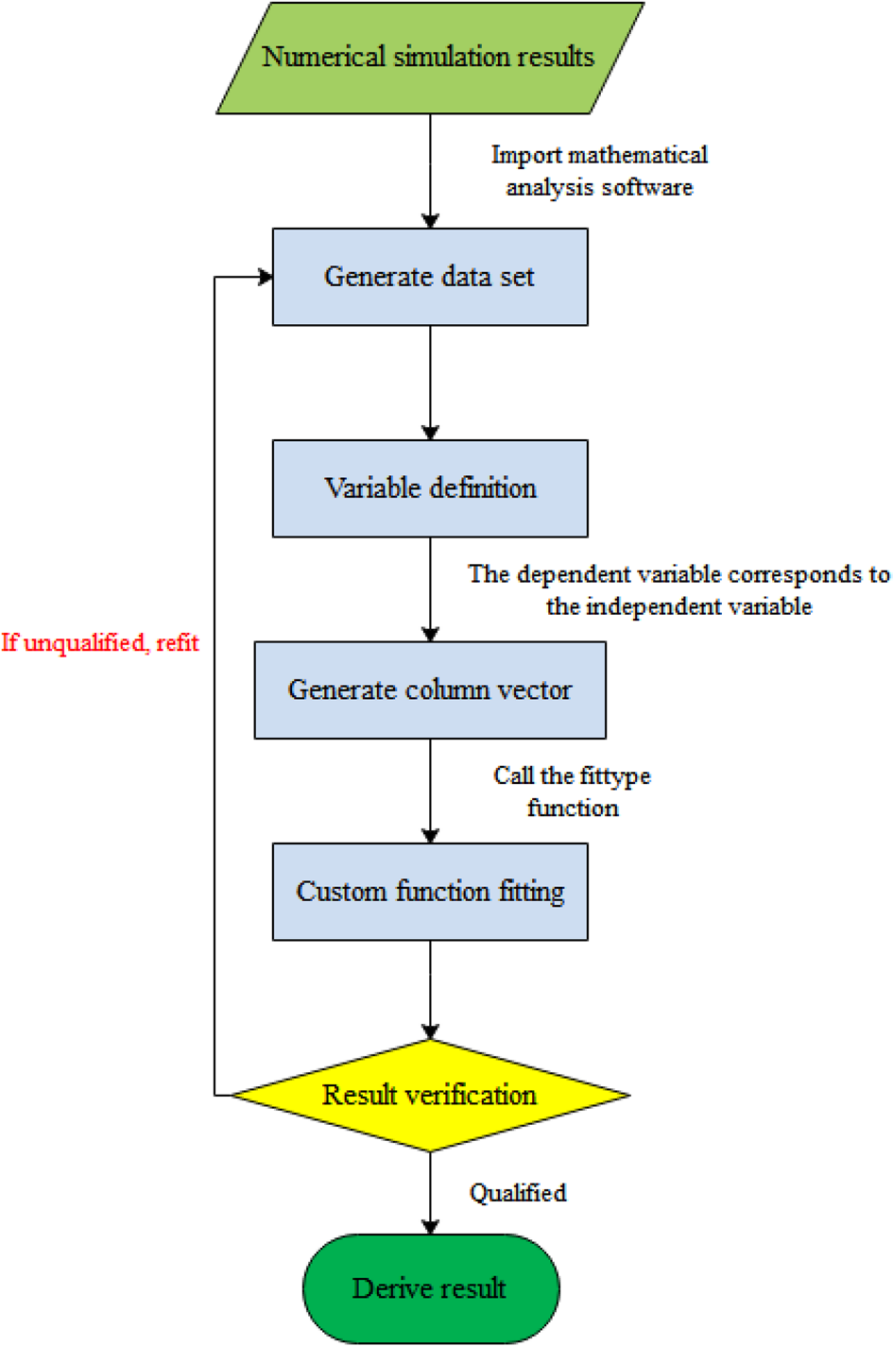
Flow chart for data fitting.

The numerical simulation results are extracted and imported into mathematical software MATLAB. The software's built-in fittype custom fitting function is then called to fit the data to Eq. (21) in order to obtain the correction coefficients *λ* and *η* for different work methods. The specific steps for data fitting are shown in Fig. 8.

**Figure 9:**
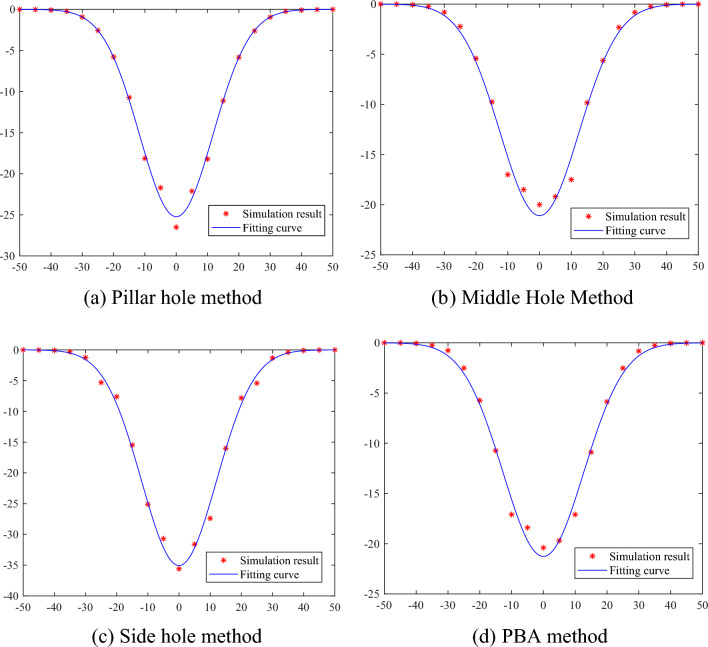
MATLAB data fit for each work method.

The default confidence level is 95%, the fitted curve obtained is shown in Fig. 9.And the values of the correction factors *λ* and *η* for each work method obtained from the fit are shown in Table 3:

**Table 3:** Table of values of λ and η for each work method.

Workmanship	*λ*	Range of values for* λ*	*η*	Range of values for *η*
Pillar hole method	−2.951	−3.05 to −2.85	−2.237	−2.30 to −2.20
Middle hole method	−2.348	−2.45 to −2.20	−2.376	−2.45 to −2.25
Side hole method	−4.57	−4.70 to −4.40	2.304	2.20 to 2.35
PBA method	−2.372	−2.40 to −2.25	−2.426	−2.50 to −2.30

As a result, different values of *λ* and *η* are taken into Eq. (21) for different construction methods to obtain the prediction equation for the settlement change of the pipe line in any overlying strata of the tunnel. To further test the applicability and accuracy of the model, the model described in this paper is now applied to the actual project.

## Project applications

### Engineering verification of the post and hole method

#### Project overview

**Figure 10:**
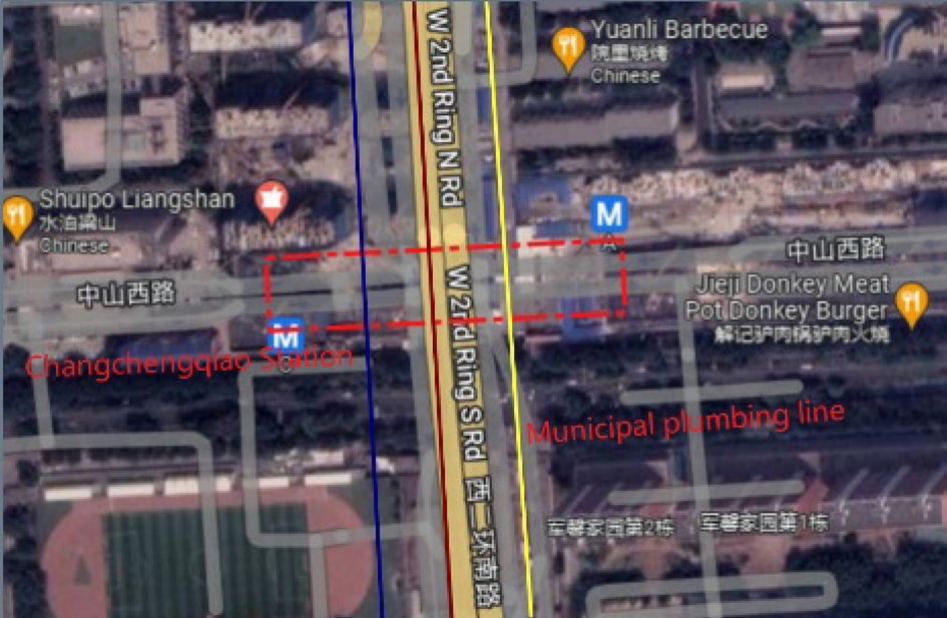
General plan of the station (image from Google Maps maps.google.com).

**Figure 11:**
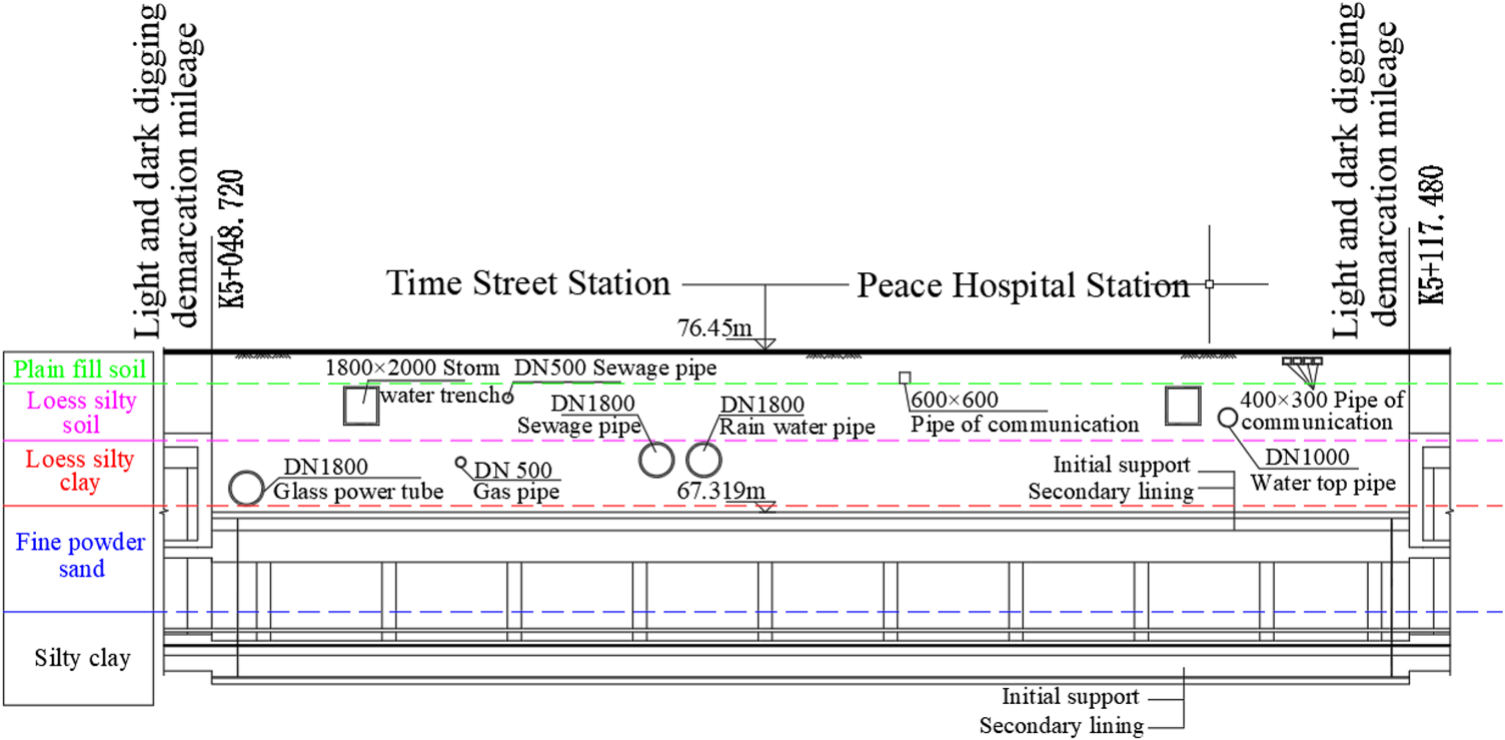
Schematic diagram of the relative position of the station and underground pipelines and the distribution of strata.

The Great Wall Bridge Station underground project is located at the crossroads of West Zhongshan Road and West Second Ring Road in Shijiazhuang, and is laid out under the road along West Zhongshan Road in an east–west direction, as shown in Fig. 10. The station is a two-storey, three-span box frame structure at both ends of the station, and a concealed single-storey, three-span structure constructed by the "pillar and hole method" in the middle section. The mileage of the concealed structure ranges from K5 + 048.72 to K5 + 117.48, with a length of 68.76 m. The central section of the concealed excavation vertically crosses several municipal pipelines, with complex surrounding conditions and high construction risks.The top of DN1800mm rainwater pipe is buried at a depth of about 5 m, with a net distance of about 3 m from the top plate of the initial support of the concealed excavation station; the top of DN1800mm sewage pipe is buried at a depth of about 5 m, with a net distance of about 3 m from the concealed excavation station; the top of DN1000mm water pipe is buried at a depth of about 3.13 m, with a net distance of about 4.8 m from the concealed excavation station. The stratigraphy of the concealed excavation station is from the top to the bottom of the ground surface: plain fill, loess-like chalk, loess-like chalky clay, fine chalky sand and chalky clay, the main body of the station crosses two strata, the upper part is chalky sand and the lower part is chalky clay. The details are shown in Fig. 11.

#### Forecast versus actual

Due to the presence of multiple overlying pipelines in the project, this section focuses on DN1800 power pipe, DN1800 rainwater pipe, and DN1000 water pipe as the primary research subjects in order to avoid duplication, and to test the accuracy and applicability of the model proposed in this paper.

**Figure 12:**
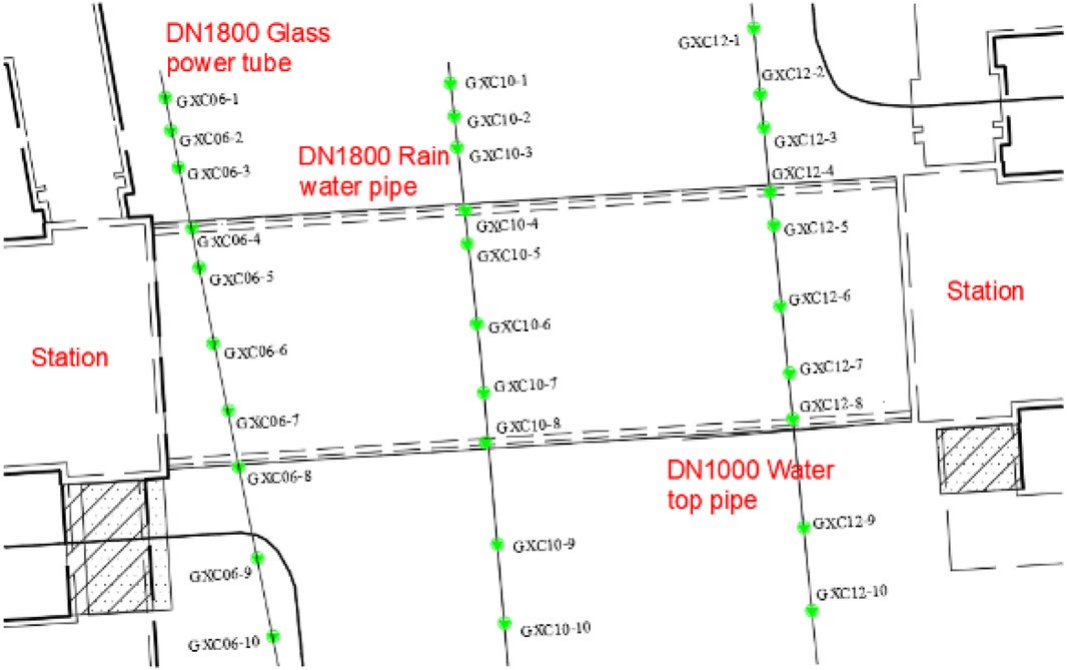
Layout of settlement monitoring points.

In order to provide timely feedback on the deformation of the pipelines during the construction process and to avoid pipeline rupture damage, a series of settlement calculation taking measurement lines were laid out for each of the overlying pipelines in the project, as shown in Fig. 12. Among them, the monitoring number of DN1800 power pipe is GXCO6, with a total of 10 measurement points; the monitoring number of DN1800 rainwater pipe is GXC10, with a total of 10 measurement points; the monitoring number of DN1000 upper water pipe is GXC12, with 10 measurement points.

**Figure 13:**
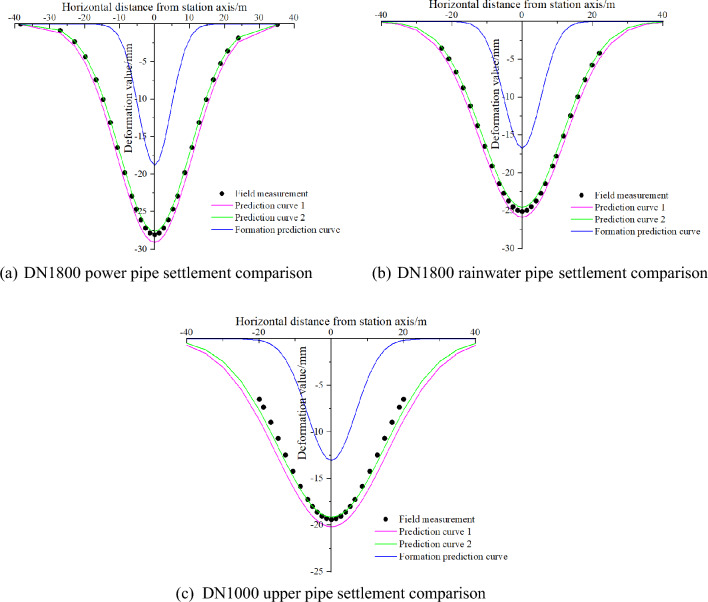
Comparison of theoretical predictions and measured data.

The project is constructed by the pillar hole method, so the coefficients *λ* of the prediction equations in this paper range from −3.05 to −2.85 and *η* from −2.30 to −2.20. *λ* is now taken as −3.05 and *η* as −2.30 and brought into the model of this paper as prediction Eq. (1); *λ* is taken as −2.85 and* η* as −2.20 and brought into the model of this paper as prediction Eq. (2); two prediction equations are used and a modified stochastic medium model for calculating ground settlement were used to predict the bottom settlement of the three pipelines and plotted together with the actual data measured in the field as follows:

The results shown in Fig. 13 indicate that the prediction equation proposed in this paper is more accurate in predicting the settlement deformation of pipelines compared to the improved stochastic medium model that directly predicts changes in ground settlement. Additionally, the two prediction curves obtained by taking the extreme values of the coefficients *λ* and *η* can roughly encompass the measured pipeline settlement curve. To better reflect the correlation between the predicted and measured data, a grey correlation analysis is utilized, calculated as follows^34,35^:

Let the parent sequence be *X*_0_ = (*x*_0_(*j*),*j* = 1,2,…,*k*) and *X*_i_ = (*x*_i_(*j*),*j* = 1,2,…,*k*) be the characteristic sequence, the grey correlation coefficient is defined as:

22 $$\begin{gathered} r(x_{0}^{{}} (j),x_{i}^{{}} (j)) \hfill \\ = \frac{{\mathop {\min }\limits_{i} \mathop {\min }\limits_{j} \left| {x_{0}^{{}} (j) - x_{i}^{{}} (j)} \right| + \rho \cdot \mathop {\max }\limits_{i} \mathop {\max }\limits_{j} \left| {x_{0}^{{}} (j) - x_{i}^{{}} (j)} \right|}}{{\left| {x_{0}^{{}} (j) - x_{i}^{{}} (j)} \right| + \rho \cdot \mathop {\max }\limits_{i} \mathop {\max }\limits_{j} \left| {x_{0}^{{}} (j) - x_{i}^{{}} (j)} \right|}} \hfill \\ \end{gathered}$$

23 $$\gamma (X_{0}^{{}} ,X_{i}^{{}} ) = \frac{1}{k} \cdot \sum\nolimits_{j = 1}^{k} {r(x_{0}^{{}} (j),x_{i}^{{}} (j))}$$

where: *ρ* is the differentiation coefficient, *ρ *∈ [0, 1]; the value of *γ*(*X*_0_, *X*_*i*_) indicates the degree of similarity between the *i*th column *x*_*i*_(*j*) and the parent sequence *x*_0_(*j*), the larger *γ*(*X*_*0*_, *X*_*i*_) is, the closer the characteristic sequence is to the parent sequence, the higher the similarity and better the correlation between the two.

The correlation between the predicted curves 1 and 2 and the measured curves was calculated using this analysis method, and the results are shown in Table 4:

**Table 4:** Table of correlation between predicted and measured curves for each pipeline.

Pipelines	Prediction curve 1	Prediction curve 2
Power pipe	0.81	0.89
Rainwater pipe	0.77	0.84
Upper water pipe	0.72	0.79

From the table, we can find that the correlation between the two prediction curves and the measured data is greater than 0.7, and some of the curves are better than 0.8. The reason why the calculated correlation is not all greater may be because prediction curves 1 and 2 are prediction curves obtained by taking the boundary value of the correction coefficient, and their correlation with the measured data is the very small value within the range of the correction coefficient taken. In practical applications, such limit cases are not usually taken, so the correlation is necessarily better than these two sets of cases in practical applications. This shows that the prediction model is able to predict the deformation of pipelines in the overlying strata of the tunnel with good accuracy for different section sizes and strata.

### Verification of other workmanship works

In order to further verify the applicability of the model in this paper, field measurement data of relevant projects were investigated and the model in this paper was used to predict the settlement of pipelines in typical research projects.

**Figure 14:**
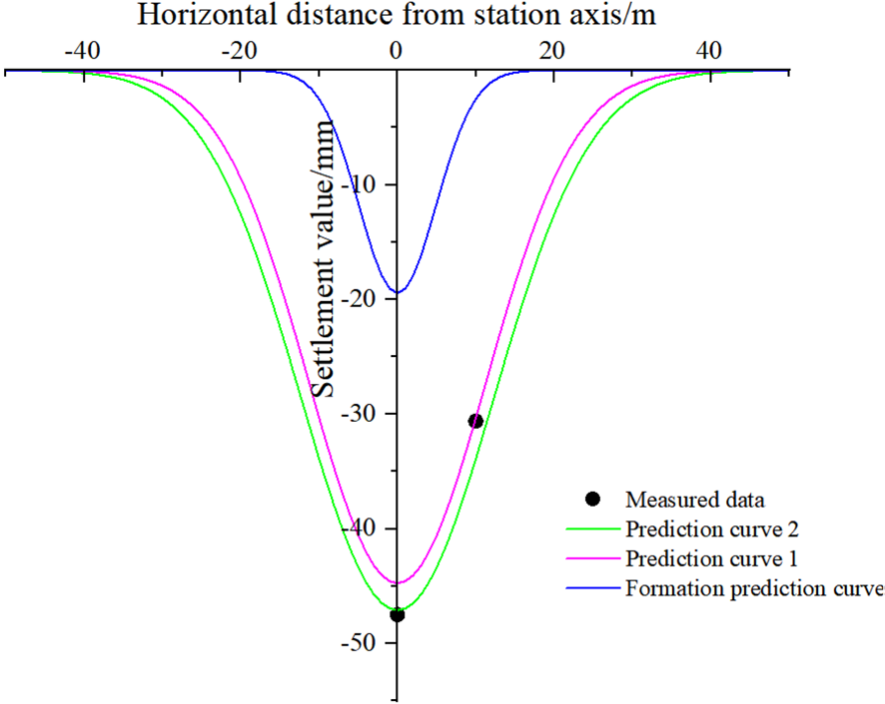
Comparison of settlement of ϕ800 cast iron pipes (predicted curve 1 shows both correction factors as very small values, predicted curve 2 shows both correction factors as very large values).

Huangzhuang Station of Beijing Metro is a cross interchange station of Metro Line 4 and Line 10, and the two stations are obliquely intersected by the PBA method of construction^36,37^; for the ϕ800 cast iron pipes above the station, the settlement is predicted using this paper's model and the calculated ground settlement model, and the predicted curves are plotted together with the measured data in Fig. 14.

From the comparison in Fig. 14, it can be found that the boundary curve of the pipeline settlement prediction model described in this paper can roughly include the measured data, and the prediction error can be controlled within ± 2 mm; as there are two tunnels underneath the pipeline using the PBA method, the correlation between the two sets of prediction curves and the measured data obtained by using grey correlation analysis is not particularly high, but The correlation between the two sets of predicted curves and the measured data is not particularly high, but it is still about 0.7, which is a good correlation, thus further demonstrating the applicability and accuracy of the model in this paper.

## Conclusion

In this paper, on the basis of the random medium theory, we summarise the experience of previous studies and modify the improved random medium theory to obtain a prediction model for predicting the deformation of pipelines in overlying arbitrary strata caused by tunnel construction, and through numerical simulations, we construct four models for the construction of metro stations commonly used at present, namely the pillar hole method, the middle hole method, the side hole method and the PBA method, and determine the correction coefficients of the pipeline deformation prediction models under different working methods The range of the prediction models under different work methods was determined, and the prediction models were validated with engineering examples, with the following main conclusions:

Based on the random medium theory and Peck's formula, the numerical models of pillar hole method, middle hole method, side hole method and PBA method were established for different construction methods, the analysis results were extracted to fit the prediction models, the range of the prediction model correction coefficients λ and η for the four common methods were obtained, and the prediction models of pipeline deformation of overlying strata caused by different construction methods of concealed underground stations were proposed.

The results of the calculation and analysis also show that the settlement of the pipeline caused by the side hole method is the largest, followed by the pillar hole method, while the settlement of the pipeline caused by the middle hole method and the PBA method is smaller. When the prediction model is applied to the actual project and the prediction results are compared with the measured data, the prediction model proposed in this paper can predict the deformation of pipelines in the stratum better and maintain a high prediction accuracy for different section sizes and different types of underground pipelines in the stratum (Supplementary Information).

### Compliance with ethical standards

This study was supported by High-Speed Rail Joint-Fund Funded Projects (U2034245) and the National Natural Science Foundation of China Youth Fund (5210082505).

## Supplementary Information

Supplementary Information.

## Data Availability

The data can be obtained from author Kaimeng Ma at mkm@my.swjtu.edu.cn.
