# Supplementary material for: Modified stochastic medium prediction model for the deformation response of concealed underground stations under existing pipelines
Source: Sci Rep. 2023 Jun 17;13:9843. doi: 10.1038/s41598-023-37067-3 (PMC10276806; doi:10.1038/s41598-023-37067-3)
Supplement: Supplementary file 1 — Supplementary Information. [file 41598_2023_37067_MOESM1_ESM.docx]

## Appendix 1

Table of symbols and their meanings

| Symbol | Symbolic meaning |
| --- | --- |
| *H* | Distance from the tunnel center to the surface |
| *Ω* | Initial section of excavation |
| *ω* | The tunnel section shrinks after completion |
| *x* | The distance of a surface location from the center of a cell |
| *W_e_(x)* | Final settlement value of the surface |
| *r(z)* | The main range of influence in the horizontal plane of the cell at depth *z* |
| *z* | Cell depth |
| *β* | Stratum main influence Angle |
| *W(x)* | Surface subsidence |
| *W_Ω_(x)* | The excavation range is the subsidence caused by *Ω* |
| *W_ω_(x)* | The excavation range is the surface subsidence caused by *ω* |
| *R* | Tunnel excavation radius |
| *u_i_(i=1,2,3)* | Convergence value of section |
| *i* | Width of settling tank |
| *S_max_* | Maximum land settlement |
| *V* | Stratum loss per unit length of tunnel |
| *V_l_* | Rate of formation volume loss |
| *D* | Tunnel diameter |
| *∆R* | Equivalent uniform shrinkage value of section radius |
| *K* | Width parameter of settling trough |
| *b* | Parameters that take into account formation properties |
| *ϕ* | The weighted average of the internal friction angles of different layers above the tunnel vault by thickness |
| *A* | Tunnel excavation area |
| *λ* | correction factor of Smax |
| *η* | correction factor of *i* |
| *X_0_=(x_0_(j),j=1,2,…,k)* | Parent sequence of grey relational degree analysis |
| *X_i_=(x_i_(j),j=1,2,…,k)* | Feature sequence of grey relational degree analysis |
| *ρ* | Differentiation coefficient |
| *γ(X_0_,X_i_)* | Sequence similarity degree |

Note: Listed in order of appearance in the text

## Appendix 2

An improved stochastic medium model for predicting pipeline settlement：

$$W(\mathrm{x})=\frac{0.798A}{i}\left(1-\sqrt{1-0.565\lambda V_{l}}\right)\exp\left(-\frac{x^{2}}{2(\eta i)^{2}}\right)$$

Table of values of λ and η for each work method

| Workmanship | Range of values for *λ* | Range of values for *η* |
| --- | --- | --- |
| Pillar hole method | -3.05 to -2.85 | -2.30~-2.20 |
| Middle Hole Method | -2.45~-2.20 | -2.45~-2.25 |
| Side hole method | -4.70~-4.40 | 2.20~2.35 |
| PBA method | -2.40~-2.25 | -2.50~-2.30 |

Symbol specification

| Symbol | Symbolic meaning |
| --- | --- |
| *A* | Tunnel excavation area |
| *i* | Width of settling tank |
| *V_l_* | Rate of formation volume loss |
| *x* | Horizontal distance from the center of the tunnel |
